# A Longitudinal Study of Emotional and Behavioral Problems among Malaysian School Children

**DOI:** 10.5334/aogh.2336

**Published:** 2019-03-07

**Authors:** Idayu Badilla Idris, Jane Barlow, Alan Dolan

**Affiliations:** 1 Department of Community Health, Faculty of Medicine, University Kebangsaan Malaysia Medical Centre, Jalan Yaacob Latiff, Bandar Tun Razak, Cheras, Kuala Lumpur, MY; 2 Department of Social Policy and Intervention, Barnett House, 32 Wellington Square, Oxford, OX1 2ER, UK; 3 Centre of Lifelong Learning, Westwood Campus, University of Warwick, Coventry, CV4 7AL, UK

## Abstract

**Background:**

Emotional and behavioral problems (EBD) or mental health problems in children and adolescents are an important public health issue, but there has been no evaluation to date of the extent of such problems in near-developed countries. This study evaluated the prevalence and stability of EBD among children in Malaysia.

**Methods:**

This research comprises a longitudinal population-based study that measured the prevalence and 6-month stability of EBD in children aged seven to eight years and thirteen to fourteen years attending public schools in Malaysia based on parents, teachers and children’s (aged 13 to 14 years) report of the Strengths and Difficulties Questionnaire (SDQ) at baseline and 6 months later.

**Findings:**

The prevalence of EBD in Malaysian school children was 9.3% for teacher-report, 8.5% for parent-report and 3.9% for child-report. There was no significance difference in the prevalence of emotional and behavioral problems over six-months for all informants, except for teacher-report Emotional and Conduct problems scores which increased significantly and child-report Total Difficulties and Emotional problems scores which decreased significantly (*p* < 0.05).

**Conclusions:**

This study shows that the prevalence of EBD among Malaysian children is almost similar to the Western countries and stable over a 6-month period. These findings suggest the need for policy makers in near-developed countries to provide services aimed at preventing EBD and treating children identified as having such problems.

## Background

Emotional and behavioral problems (emotional and behavioral disabilities or EBD) or mental health problems in children and adolescents have increased in many countries over the past decade [1,2,3]. EBD that remains untreated may result in long-term problems such as withdrawal, anxiety and depression, and in the case of externalizing conditions such as conduct disorder, may ultimately result in antisocial psychiatric disorder (APD) [4]. For example, one longitudinal follow-up study of children attending a child guidance clinic, found a relationship between behavioral problems in childhood and adult antisocial personality disorder (APD) [5]. Similarly, Fergusson, Boden and Horwood [6] found that conduct disorders are associated with increased risk of criminal behavior, mental health problems, substance abuse, relationship and health problems in later adolescence and early adulthood. Childhood behavior problems have also been found to increase the risk of injuries in children and adolescents [7,8,9]. EBD is also associated with educational problems such as school dropout, and wider social problems such as domestic violence, unwanted pregnancies and physical health problems [10]. Conduct problems that begin in early childhood have a higher stability and such children are at more risk of developing problems later in life compared with adolescent-onset conduct problems [11,12].

Although these problems can be detected in early childhood through screening using validated psychological questionnaires with multiple informants [10], many children with EBD are not identified or referred to mental health facilities for treatment [13,14,15]. Lavigne et al. [16] found that 51.7% of preschool children with EBD in Paediatric Primary Care settings in the US were not given appropriate treatment. The Ontario Child Health Study also found that although almost one in five children was diagnosed with mental health or psychiatric problems, five out of six of these children did not receive treatment [17].

Many studies of the epidemiology of child and adolescent EBD have been conducted over the past 30 years [18] with the aim of monitoring the burden of disease and for the purpose of preventing the on-set of further disease, and establishing appropriate interventions and mental health services for this age group in the community [19,20]. A review of 52 studies [21], found that the prevalence estimates of EBD ranged from approximately 1% to nearly 51% with a mean of 15.8% in Western countries. Median rates were observed to be 8% for pre-schoolers, 12% for preadolescents and 15% for adolescents.

Most of these studies, however, have been carried out in the United States and United Kingdom, with few studies of child EBD having been conducted in developing countries [22]. This may be due to problems in defining emotional and behavioral problems, a focus on the increasing prevalence of other important clinical problems such as infectious diseases (e.g. malaria, low respiratory infections, HIV and other new emerging infectious diseases) [23], as well as a lack of professional providers of mental health facilities in developing countries [24,25]. However, studies on child EBD are becoming more common in Eastern countries, due to an increasing awareness that rapid urbanization in these countries has led to an increase in such problems [24].

In addition, there has been little research conducted on child’s emotional and behavioral problems in Malaysia. One of the few population-based studies that measured the prevalence of mental health problems among rural Malaysian children was undertaken in 1993, and it was estimated that 6.1% of these children had some form of mental health disorder [27]. Furthermore, there appears to have been no longitudinal studies assessing the stability of these problems over time in Malaysia. This paper reports the findings of a population study of the prevalence of EBD according to the five subscales in the Strengths and Difficulties Scores (SDQ) in children in Malaysia when rated by parents, teachers and children including variation by age and gender. This study also examined the stability of these problems between two time points (i.e. 6 months). We hypothesize that there is an association between the children’s age and gender, and their Emotional, Conduct, Hyperactivity and Peer Problems. We also hypothesize that EBD among children in Malaysia is stable over time.

## Methods

### Sample

This research was carried out among parents and teachers of children as well as the respective children from selected schools, in the areas of Petaling and Klang in Selangor, two of the most populous and developed city’s in Malaysia. The samples were randomly selected from a list of 161 primary and 95 secondary schools in both districts in June 2012. A total of six primary and three secondary schools were randomly selected from the above list. Children (and their respective parents and teachers) who were selected from the primary schools, were all in Year 1 and 2 (7 to 8 years old) while those who were selected from the secondary schools were all in Form 1 and 2 (13 to 14 years old). The research was carried out by using stratified multistage cluster sampling. Sample size was calculated using the highest and lowest prevalence estimates of emotional and behavioral problems among children in Asia which were 12% and 34% respectively [25,26]. Using this prevalence rate, the calculated sample size was 495, using α = 0.05 and β = 0.95. Most children were male (52%) and their mean age was 13.5.

### Data collection tool

The parents, teachers and respective children (aged 13 to 14 years only) from the selected schools completed the self-administered Strengths and Difficulties Questionnaire (SDQ). The SDQ has been used widely in research across the world, and has been translated into more than 40 languages, and can be downloaded free from the internet [27]. The SDQ has also been translated into the Malay Language, which is the main language used by participants in this research, although the children’s version is not translated or freely available, and thus had to be translated as part of this study. The SDQ is a brief screening instrument developed by Goodman (1997) that is used to assess emotional and behavioral problems in children and adolescents. It can be completed in five to ten minutes and comprises 25 core items, which include 14 Difficulties items, 10 Strengths items and 1 Neutral item. Each item is answered on a three-point Likert scale as follows: ‘true’, ‘somewhat true’ and ‘certainly true’. The SDQ questionnaire can be completed by parents of 3 to 16-year-old children as well as by children aged 11 to 16 years old. The 25 items can be divided into 5 subscales that include Emotional, Conduct, Hyperactivity, Peer-problems and Pro-social skills. The first 4 symptoms when added together would yield Total Difficulties Scores (TDS) which is based on 20 items. Children will be classified as normal by parents when the Total Difficulties Scores were between 0–13, borderline, i.e. when scores were between 14–16 and abnormal when the TDS were between 17–40. In this study, the Chronbach’s alpha of this questionnaire in Malay, was found to be more than 0.7 based on parent’s, teacher’s and children’s score. Factor analysis showed a 5-factor solution, which was consistent with many other psychometric evaluations of the SDQ in other languages.

### Response rate

A total of 900 questionnaires were distributed, of which 501 were returned by the parents. Six of the questionnaires were discarded due to incomplete data, leaving 495 questionnaires with complete data, giving a response rate of 55%. In addition, 430 questionnaires were returned by the teachers. A total of 423 questionnaires had complete data, giving a response rate of 47% for teachers (8 questionnaires were discarded due to incomplete data). Similarly, 154 children from the secondary schools completed the questionnaires (seven students were absent on the day of data collection) giving a response rate of 51.3%.

Of the 495 parents who completed the questionnaires at baseline, a total of 330 parents also returned the questionnaires at six months. Three hundred and twenty-three had complete questionnaires. In addition, 311 teachers returned questionnaires at the six-month follow-up, 304 of which were complete, One hundred and thirteen secondary school children completed the questionnaires at follow-up.

### Procedure

Approval for this study was provided by the Biomedical Research Ethics Committee (BREC) University of Warwick, the Economic Planning Unit and Ministry of Education in Malaysia, and the Department of Education in Selangor, Malaysia. Prior to distribution of questionnaires, the translation of the child version of SDQ as well as assessment of face validity during a pre-test was carried out. A full translation was undertaken, although the only difference between the parent’s/teacher’s version and the children’s version involved grammatical alterations from the third person to the first person. A letter of invitation to mothers or caregivers along with an information sheet, parental consent form and a copy of the SDQ was given to children from the randomly selected schools who were asked to take these questionnaires home for completion by their parents. If the parents agreed to take part in this research, mothers/caregivers were asked to return the consent form and questionnaires in an envelope, to the school teachers, and these were subsequently collected by the researcher. Sealed envelopes were used to maintain confidentiality. The teachers of the children were also invited to complete the questionnaires on the respective children during school, where parental consent for this was given. The respective child (for whom the parent had consented) in the secondary schools, were invited to complete the questionnaire about their emotional and behavioral problems (child-report of the SDQ) in the school setting, on a planned date. The SDQ questionnaires were completed again 6 months later, by the same parents, teachers and children.

### Data analysis

Statistical analysis was carried out using SPSS version 22. Categorical data for Total Difficulties Scores and each subscales were analysed using the chi square test. Means and standard deviations were also calculated for each subscale and are presented according to age and gender. The stability of the scores for all subscales was then assessed by comparing the data at the two time points using a t-test.

## Results

Table 1 shows the socio-demographic characteristics of respondents in primary schools compared with respondents in secondary schools. Overall, a majority of the respondents were Malays, in both the primary and secondary schools. This was anticipated because public schools are mainly enrolled by Malays as opposed to the Chinese or Indians who prefer to send their children to vernacular schools. Table 1 also shows that there were more Malay children in the secondary schools compared with primary schools (86.5% vs. 95.7%) and this difference is significant (*p* = 0.003). More children were male (52%) but the difference across primary and secondary schools, was not significant (*p* = 0.378).

**Table 1:** Comparisons of socio-demographic characteristics of mothers/caregivers and children according to primary and secondary schools in the prevalence study.

Characteristics	Primary	Secondary	*p* (significance level)

Respondent			
Mothers	307 (91.9%)	143 (88.8%)	0.450
Fathers	24 (7.7%)	16 (9.9%)	
Other family members	1 (0.3%)	1 (0.6%)	
Others	2 (0.6%)	1 (0.6%)	
**Sex**

Male	178 (53.3%)	79 (49.1%)	0.378
Female	156 (46.7%)	82 (80.9%)	
**Race**

Malay	289 (86.5%)	154 (95.7%)	0.003***
Chinese	3 (0.9%)	3 (1.9%)	
Indian	40 (12.0%)	4 (2.5%)	
Sikhs	2 (0.6%)	0 (0%)	
**Family Type**

Single	1 (0.3%)	0 (0%)	0.863
Married	319 (95.5%)	153 (95.0%)	
Divorce	8 (2.4%)	4 (2.5%)	
Death
(Father)	6 (1.8%)	4 (2.5%)	
**Maternal/Female’s Carer Age**

20–29	12 (3.6%)	0 (0%)	0.000***
30–39	214 (64.1%)	54 (33.5%)	
40–49	103 (30.8%)	90 (55.9%)	
50–59	5 (1.5%)	16 (9.9%)	
60–69	0 (0%)	1 (0.6%)	
**Maternal Education**

No Education	1 (0.3%)	1 (1.9%)	0.000***
Primary	14 (4.2%)	1 (0.6%)	
Secondary	175 (52.4%)	126 (78.3%)	
Tertiary	141 (42.2%)	30 (18.6%	
No information	3 (0.9%)	1 (0.6%)	
**Maternal Working Status**

Working	228 (68.3%)	86 (53.4%)	0.004***
Not working	103 (30.8%)	74 (46.0%)	
No information	3 (0.9%)	1 (0.6%)	
**Child’s carer**

Parents/Caregivers	127 (38%)	105 (65.2%)	0.000***
Other family members	74 (22.2%)	27 (16.8%)	
Babysitters	81 (24.3%)	16 (9.9%)	
Maid	52 (15.6%)	13 (8.1%)	
**Monthly family Income**

Low (<GBP 300)	47 (14.1%0	30 (18.6%)	0.214
Middle (GBP 300–GB1200)	187 (56.0%)	93 (57.8%)	
High (>GBP 1200)	100 (29.9%)	38 (23.6%)	
**Number of siblings**

Less than 4	203 (60.8%)	65 (40.4%)	0.000***
4–6	119 (35.6%0	79 (49.1%)	
More than 6	12 (3.6%)	17 (10.6%)	

* significant *p*-value, *p* < 0.05. ** significant *p*-value, *p* < 0.01. *** significant *p*-value, *p* < 0.001.

There were significant differences in terms of the maternal carer’s age, with mothers of children in the 7–8-year age group being younger compared with mothers of children in the 13–14-year age group as would be expected (*p* < 0.000). There were also significant differences in maternal education (*p* < 0.000) and maternal working status (*p* = 0.004) in terms of there being more mothers of children in primary school who were working and who had tertiary education compared to mothers of children in secondary schools.

Table 2 summarizes the findings from the parent, teacher and child-reports of the SDQ for the Total Difficulties Scores as well as the prevalence of the other five subscales (Emotional problems; Conduct problems; Hyperactivity problems; Peer relations; Pro-social skills) at baseline. This table shows that teachers rated the most children (9.3%) as having problematic behavior (Total Difficulties Problems), followed by parents (8.5%) and children (3.9%).

**Table 2:** Prevalence of parent, teacher and childreports of emotional and behavioral problems at baseline.

Scores	Parent (n = 495) n (%)	Teacher (n = 432) n (%)	Children (n = 154) n (%)	*p* level

TDS				
Normal	410 (82.8)	338 (78.2)	133 (86.4)	0.155
Borderline	43 (8.7)	54 (12.5)	15 (9.7)	
Abnormal	42 (8.5)	**40 (9.3)**	6 (3.9)	
**Emotional**				

Normal	409 (82.6)	398 (92.1)	135 (87.7)	0.000***
Borderline	45 (9.1)	17 (3.9)	13 (8.4)	
Abnormal	**41 (8.3)**	17 (3.9)	6 (3.9)	
**Conduct**

Normal	361 (72.9)	364 (84.3)	135 (87.7)	0.001**
Borderline	78 (15.8)	42 (9.7)	13 (8.4)	
Abnormal	**56 (11.3)**	26 (6.0)	6 (3.9)	
**Hyperactivity**

Normal	435 (87.9)	375 (86.8)	136 (87.0)	0.901
Borderline	35 (7.1)	32 (7.4)	11 (7.1)	
Abnormal	25 (5.1)	**25 (5.8)**	**9 (5.8)**	
**Peer Problem**

Normal	312 (63.0)	290 (67.1)	125 (81.7)	0.000***
Borderline	91 (18.4)	102 (23.6)	26 (16.9)	
Abnormal	**92 (18.6)**	40 (9.3)	3 (1.4)	
**Pro Social**

Normal	425 (85.9)	305 (70.8)	118 (76.6)	0.000***
Borderline	47 (9.5)	75 (17.4)	22 (14.3)	
Abnormal	23 (4.6)	**52 (12.0)**	14 (9.1)	

TDS – Total Difficulties Scores. * significant *p*-value, *p* < 0.05. ** significant *p*-value, *p* < 0.01. *** significant *p*-value, *p* < 0.001.

A chi-square test was then used to examine the relationship between parent, teacher and child scores within each category. Using Bonferroni adjusted alpha level of 0.005, we found that the Total Difficulties Scores between the raters were not significantly different. However, for the parent, teacher, and children reports, parents produced the highest abnormal scores for Emotional, Conduct and Peer problem domains (*p* < 0.000).

Table 3 shows the means and standard deviations for the Total Difficulties scores and all sub-scales of the SDQ according to gender at baseline. The Total Difficulties scores was significantly higher for boys based on teacher reports (*m* = 9.3, *p* = 0.007) and much higher for girls based on children reports (*m* = 11.6, *p* = 0.045). Emotional problems were higher for girls based on child-report data and this difference was significant (*m* = 3.4, *p* = 0.002). There were also more Hyperactivity problems for boys based on parent and teacher reports (*m* = 3.4, *p* = 0.001; *m* = 3.4, *p* < 0.000) respectively. Peer problems were also higher for boys based on parent reports (*m* = 2.4, *p* = 0.005) and this difference was also significant.

**Table 3:** Mean and standard deviation of emotional and behavioral problems by gender at baseline.

Scores	Parent (n = 495) *m* (sd)			Teacher (n = 432) *m* (sd)			Children (n = 154) *m* (sd)		

	M	F	*p* level	Male	F	*p* level	Male	F	*p* level

**Total Difficulties**	9.0 (4.9)	8.4 (5.1)	0.180	9.3 (5.1)	8.0 (5.0)	0.007**	10.1 (4.3)	11.6 (4.9)	0.045*
**Emotional**	1.6 (1.7)	1.9 (1.9)	0.057	1.6 (1.8)	1.6 (1.8)	0.963	2.5 (1.7)	3.4 (2.2)	0.002**
**Conduct**	1.7 (1.5)	1.7 (1.5)	0.543	1.4 (1.4)	1.2 (1.4)	0.114	2.4 (1.7)	2.1 (1.3)	0.199
**Hyperactivity**	3.4 (2.1)	2.7 (1.9)	0.001**	3.4 (2.0)	2.6 (2.1)	0.000***	3.1 (1.8)	3.6 (2.0)	0.061
**Peer Relation**	2.4 (1.5)	2.1 (1.4)	0.005	2.9 (1.5)	2.6 (1.5)	0.076	2.2 (1.4)	2.5 (1.4)	0.231
**Pro-Social**	7.5 (1.9)	7.5 (1.8)	0.791	6.7 (2.2)	7.3 (2.2)	0.007**	6.5 (1.7)	6.9 (1.7)	0.109

Independent T Test. * significant *p*-value, *p* < 0.05. ** significant *p*-value, *p* < 0.01. *** significant *p*-value, *p* < 0.001.

Table 4 shows the prevalence of EBD for Malaysian school children according to age at baseline. This table shows that there were higher Total Difficulties scores (*m* = 9.2, *p* = 0.002), Conduct (*m* = 1.8, *p* = 0.04) and Hyperactivity problem scores (*m* = 3.3, *p* < 0.00) for younger children based on parent-report data compared with teacher or child-report data. Teacher-reports, however, show significantly higher problems for older children for Total Difficulties scores (*m* = 9.8, *p* < 0.00), Conduct (*m* = 1.7, *p* < 0.00), Hyperactivity (*m* = 3.3, *p* = 0.02) and Peer problems subscales (*m* = 3.0, *p* < 0.01), when compared to parent’s and children’s reports.

**Table 4:** Mean and standard deviation of emotional and behavioral problems by age at baseline.

	Parents (n = 495)			Teachers (n = 432)		

	Primary n (%)	Secondary n (%)	*p* level	Primary n (%)	Secondary n (%)	*p* Level

TDS

Normal	268 (65.2%)	143 (34.8%)	0.005***	220 (65.3%)	117 (34.7%)	0.581
Borderline	29 (67.4%)	14 (32.6%)		34 (61.8%)	21 (38.2%)	
Abnormal	37 (90.2%)	4 (9.8%)		23 (57.5%)	17 (42.5%)	
**Emotional**						

Normal	271 (66.3%)	138 (33.7%)	0.410	259 (65.1%)	139 (41.2%)	0.132
Borderline	32 (71.1%)	13 (28.9%)		7 (41.2%)	10 (58.8%)	
Abnormal	31 (75.6%)	10 (24.4%)		11 (64.7%)	6 (35.3%)	
**Conduct**						

Normal	236 (65.4%)	125 (34.6%)	0.137	245 (67.5%)	118 (32.5%)	0.001**
Borderline	54 (69.2%)	24 (30.8%)		17 (39.5%)	26 (60.5%)	
Abnormal	44 (78.6%)	12 (21.4%)		15 (57.7%)	11 (42.3%)	
**Hyperactivity**						

Normal	285 (65.5%)	150 (34.5%)	0.042**	238 (63.5%)	137 (36.5%)	0.210
Borderline	29 (82.9%)	6 (17.1%)		19 (59.4%)	13 (40.6%)	
Abnormal	20 (80.0%)	5 (20.0%)		20 (80.0%)	5 (20.0%)	
**Peer-Problem**						

Normal	205 (65.7%)	107 (34.3%)	0.513	200 (69.0%)	90 (31.0%)	0.011*
Borderline	63 (69.2%)	28 (30.8%)		55 (53.9%)	47 (46.1%)	
Abnormal	66 (71.7%)	26 (28.3%)		22 (55.0%)	18 (45.0%)	
**Pro-Social Skill**						

Normal	285 (67.1%)	140 (32.9%)	0.887	188 (61.6%)	117 (38.4%)	0.102
Borderline	33 (70.2%)	14 (29.8%)		49 (65.3%)	26 (34.7%)	
Abnormal	16 (69.6%)	7 (30.4%)		40 (76.9%)	12 (23.1%)	
**Total**	334	161		277	155	

TDS = Total Difficulties Score. Independent T Test. * significant *p*-value, *p* < 0.05. ** significant *p*-value, *p* < 0.01. *** significant *p*-value, *p* < 0.001.

In terms of stability, we defined EBD as being stable over time, if there was no significant increase or decrease in scores between Time 1 and Time 2. Table 5 shows that the mean Total Difficulties scores and other subscale scores for parents were steady (not significantly increased or reduced) over the 6-month period. This was also the case for teacher’s Total Difficulties Scores, Hyperactivity and Peer problem subscale scores. However, there was significance increase in teacher-report Emotional and Conduct problems scores and significant decrease in child-report Total Difficulties and Emotional problems scores (*p* < 0.05). Overall, these results suggest that the mean scores are stable and maintained over time based on parent, teacher and children’s reports.

**Table 5:** Mean differences across time for parent, teacher and child report scores at baseline and 6-month follow-up.

Respondent Scores	Time 1		Time 2		*P* level

	Mean	Standard Deviation	Mean	Standard Deviation	

Parent					
TDS	8.73	4.887	8.64	5.147	0.765
Emotional Problem	1.76	1.785	1.77	1.787	0.939
Conduct Problem	1.69	1.446	1.55	1.434	0.146
Hyperactivity Problem	3.00	2.016	2.99	2.054	0.921
Peer Problem	2.28	1.433	2.33	1.541	0.641
Pro-Social Skill	7.41	1.832	7.24	2.000	0.164
**Teacher**					

Total Difficulties Scores	8.42	4.768	8.70	5.054	0.437
Emotional Problem	1.51	1.741	1.92	1.898	0.006**
Conduct Problem	1.14	1.269	1.64	1.771	0.000****
Hyperactivity Problem	2.85	2.229	3.12	2.259	0.072
Peer Problem	2.57	1.511	2.70	1.652	0.187
Pro-Social Skill	7.41	2.196	7.05	2.061	0.012
**Children**					

Total Difficulties Scores	11.23	4.685	9.93	4.360	0.002**
Emotional Problem	3.11	1.995	2.41	1.891	0.000***
Conduct Problem	2.23	1.469	1.97	1.401	0.055
Hyperactivity Problem	3.43	1.911	3.15	1.808	0.126
Peer Problem	2.46	1.447	2.41	1.363	0.716
Pro-Social Skill	6.83	1.540	6.58	1.797	0.165

TDS = Total Difficulties Score. Independent T Test. * significant *p*-value, *p* < 0.05. ** significant *p*-value, *p* < 0.01. *** significant *p*-value, *p* < 0.001.

## Discussion

The results of the prevalence study showed abnormal Total Difficulties scores for teacher reports (i.e. 9.3%), followed by parent (i.e. 8.5%) and child reports (3.9%). This suggests that the range of children rated as having EBD by all informants were within the range defined by the original cut-off points (i.e. 10% of children in the UK were defined as having abnormal scores) [28]. A review by Srinath, Kandasamy and Golhar [25] found the prevalence of child and adolescent EBD to be around 10-20% in 11 countries in Asia (i.e. Bangladesh, Afghanistan, China, Israel, Vietnam, Saudi Arabia, India, Yemen, Pakistan, Singapore and India).

In terms of gender differences, the results of this study are consistent with other studies, which also suggest that boys were more likely to have higher behavioral problems scores [15,27] and are more likely to have Conduct and Hyperactivity problems but less likely to have Emotional problems [16,29,30,31,32].

In terms of EBD differences according to age, as reported by parents, this result is also consistent with the findings of other studies. For example, Van Widenfelt, Goedhart, Treffers and Goodman [33] found a significant declining trend with age in Total Difficulties scores and Emotional problems subscale scores based on parent-report data. Van der Ende, Verhulst, Tiemeier [34] also found a decrease in Total Difficulties scores with age, when reported by parents and children. The higher reporting of problems by teachers may reflect the greater difficulty they experienced in managing such behavior in older children in this country.

The findings of this study also suggest that problems were mostly stable across all informants. Behavior was more unstable with reference to internalizing (i.e. Emotional) problems based on two informant’s reports (i.e. teacher and children) compared with externalizing (Conduct) problems, which significantly increased over time based on teacher reports only. Hyperactivity problems were stable across all informants. Significant increases in mean scores (i.e. Emotional and Conduct problems) as reported by teachers, may be due to attrition among teachers between the two time points. The fact that children showed a significant decrease in mean scores across Total Difficulties and Emotional scores could be due to the small number of children who were old enough to complete the questionnaires. Furthermore the interval between scores at Time 1 and Time 2 was short (i.e. 6 months only) and this could explain some scores that were not stable (significant increase and decrease) within 6 months.

Overall the scores were stable across time and this was consistent with other studies which show that emotional and behavioral problems among children are stable across time [35,36]. Most studies assessing the stability of emotional and behavioral problems have been conducted in the West, and we identified one study that had been conducted in a developing country (e.g. Brazil) [37], which also showed that externalizing behavior was more stable than internalizing behavior. However, comparison of the stability of EBD across studies is hindered by a range of problems including cross-cultural differences, methodological differences, and variation in the duration of time between which measurements of psychopathology are taken [38].

This study suggests that EBD among Malaysian children are similar to the prevalence of these problems in developed countries. Although Malaysia is not a fully developed country, it is building towards an increasingly modernized urban nation, even though major parts of the country still comprise large suburban and rural regions. This modernisation process has resulted in rapid social changes that may have contributed to the development of EBD, as has occurred in some developed countries.

One of the strengths of this study was the large number of participants who took part, which will have increased the validity of the results. These participants were selected using stratified random sampling, which contributed to an equal probability of eligible participants being selected [39]. The primary outcome instrument was also validated, thus ensuring acceptability among the Malaysian community.

Furthermore, to our knowledge, this is a first longitudinal research on EBD among children in Malaysia, and one of the few longitudinal studies that has been conducted on EBD among children from a low and middle-income Eastern country.

The findings of this study have some limitations. Perhaps most importantly, this study only included children aged 7 to 8 and 13 to 14 years from selected schools in an urban setting. Future studies should include a wider age range of children. A further limitation is the use of the SDQ, which is not sufficiently comprehensive to measure or capture the full range of child mental health problems. Although the results of this study point to the stability of EBD between baseline and six months, the results should be interpreted with caution for a number of reasons. The combination of two age-groups (i.e. 7 to 8 years and 13 to 14 years) could possibly have introduced a selection bias in terms of predicting the overall prevalence. For example, earlier longitudinal studies found that externalizing behavior decreased with age [40] whilst internalizing behavior which was initially stable in early childhood, particularly among girls [41], escalated with increasing age. Future studies should focus on a single age group to ensure that the developmental trajectory in these children is accurately identified. Future studies should also incorporate a longer time period between baseline and follow-up to evaluate whether the stability identified continues over longer durations of time.

Despite these limitations, this research has made a significant contribution to our knowledge about EBD in Malaysian children. The findings in terms of the stability of these problems over time, has highlighted the importance of identifying and dealing with these problems early.

## Conclusion and Future Research Direction

This research has a number of policy implications. First, the findings of this research suggest that almost 10% of children were screened to have EBD. This study also suggests the stability of EBD among children in Malaysia after a six-month period. These findings are similar to those for Western countries, and there is as such a need for policy makers to monitor the prevalence and stability of EBD to assess the public health burden among children in Malaysia.

Second, this research has highlighted a potential role for the secondary prevention of EBD among children and adolescents in Malaysia. This suggests the importance of detecting these problems early in order to provide appropriate management and thereby preventing these problems from becoming more severe in later childhood.

## Data Accessibility Statement

The data will be available upon request.
